# Benevolent Childhood Experiences and Depressive Symptoms Among Chinese Undergraduates: A Moderated Mediation Model Examining the Roles of Uncertainty Stress and Family Relationship

**DOI:** 10.3389/fpubh.2021.757466

**Published:** 2021-12-16

**Authors:** Caiyi Zhang, Wei Wang, Yifei Pei, Ying Zhang, Chenlu He, Jingjing Wang, Xiuyin Gao, Hao Hou

**Affiliations:** ^1^Department of Medical Psychology, Second Clinical College, Xuzhou Medical University, Xuzhou, China; ^2^Department of Psychiatry, The Affiliated Xuzhou Oriental Hospital of Xuzhou Medical University, Xuzhou, China; ^3^Department of Community and Health Education, School of Public Health, Xuzhou Medical University, Xuzhou, China

**Keywords:** benevolent childhood experiences, depressive symptoms, uncertainty stress, family relationship, undergraduates

## Abstract

**Background:** The evidence on the association between benevolent childhood experience (BCE) and depressive symptoms in students is complex. This study aims to explore the underlying mediation mechanism of BCE toward depressive symptoms and whether this link was moderated by the family relationship among Chinese undergraduates.

**Methods:** From March 2021 to May 2021, a cross-sectional study was conducted in China, and 1821 undergraduates were recruited in this study. Participants were asked to complete a self-reported electronic questionnaire. The software SPSS PROCESS macro was used to test the mediation and mediated moderated modeling analysis.

**Results:** Mediation analysis indicated that uncertainty stress (US) partly mediated the link between BCE and depressive symptoms (indirect effect = −0.47, 95% bootstrap CI = −0.55, −0.39). The indirect effect of the US accounted for 39.63% of the total variance in depression. Moderation analysis indicated that the association between the US and depressive symptoms was significantly modified by family relationships (interact effect = −0.019, *P* < 0.001). An integrative moderated mediation analysis indicated that the indirect effect from BCE to depressive symptoms through the US was also moderated by family relationships (interact effect = −0.012, *P* = 0.014).

**Conclusion:** Uncertainty stress plays a key role in bridging BCE and depressive symptoms while the family relationship can buffer the impact of the US on depressive symptoms among Chinese undergraduates. Enhancing tolerance of uncertainty and improving family relationships are needed to protect undergraduates from depressive symptoms.

## Introduction

### The Depressive Symptoms of Undergraduate Students as a Growing Concern

Depressive disorder is a worldwide problem, as well as the most common mental health problem. The existing study indicated that depressive symptoms with a lifetime prevalence of 16.2% and a one-year prevalence of 6.6% among the general population (1). A systematic review showed that about 19.6–30.6% (2–4) of undergraduates suffered from depressive symptoms, which is higher than the prevalence in the general population (1). A meta-study indicated that all of the gender, family origin, academic grade, only-child, ethnic group, education of parents, left-behind experiences on childhood (3), and economic condition (5) are risk factors of Chinese college students. Students suffering from depressive symptoms may adversely affect their academic performance (6), quality of life (6) and even lead to suicidal ideation (7).

‘Adverse childhood experiences (ACE) have been defined as ‘potentially traumatic events or chronic stressors that occur before the age of 18 and are uncontrollable to the child' (8), such as sexual abuse, emotional abuse, and so on, which has a positive association with depressive symptoms (9–11). However, ACE is not the only form of early experience that has long-term associations with mental health outcomes (12), the impact of benevolent childhood experiences (BCE) on depressive symptoms was also gradually focused on by many researchers (13).

### BCE and Depressive Symptoms: A Confirmed Link

Contrary to the early negative effects of ACE, BCE represents the positive early experiences under 18 years old (14), including growing up with at least one safe caregiver, having one or more close friends, and having a predictable home routine, etc. (13, 14). To date, most studies related to BCE were carried out in developed countries (15–17), while there are no studies conducted in Asia. Influenced by traditional Chinese cultural concepts and parents' work pressure, Chinese children receive more intergenerational education (18), which makes their BCE worth watching. Findings suggested that BCE may have lifelong consequences for mental health (19) and physical health (15, 20). One cross-sectional study shows that the high level of BCE can buffer the negative effects on the mental health caused by ACE (21). Moreover, other studies indicated that BCE can also predict better adult mental health dependently (13, 14, 19, 21). Although these studies have confirmed the association between BCE and depressive symptoms, however, the underlying mechanisms underlying the association between childhood experiences and adult mental health are multifactorial and complex. So far, little empirical research has been done to explore the underlying mechanism of how BCE is related to depressive symptoms among undergraduates in China.

### BCE, US, and Depressive Symptoms: A Mediation Pathway

Uncertainty stress (US) refers to the stress caused by the condition of being unsure about someone or something (22). As we all know that university students are more prone to experience high levels of uncertainty stress (23), not only including future uncertainty but also current uncertainty, for example, rapid socio-economic transition, increased job competition, immature social values, and feelings of social anomie (24, 25), which are collectively known as uncertainty stress (US). China is now one of the world's fastest-growing economies (26), many policies were also changed in recent years in China. For example, the one-child policy has been abolished by the Chinese government (27), China is still expanding enrollment at universities (28). All these changes not only reflect a dramatic change in the social environment but also may have an impact on the psychological characteristics of university students in China (3). The rapid change of the social environment and the fierce competition for jobs and the uncertainty of the future all lead to the Chinese and Chinese college students becoming the biggest victims of the pressure of uncertainty stress (29, 30).

Research indicated that uncertainty cues can arouse higher stress than certainty cues (31). Due to the traditional culture, Chinese college students are more intolerant of ambiguous states and regard uncertainty as a threatening and unacceptable presence (32, 33). A cross-sectional study found that Chinese university students who suffer from US (19.6%) are higher than life stress (11.5%) (25), and uncertainty stress had a more negative influence and adverse consequences on college students' mental health than life stress (34), which might be a unique precursor to depressive symptoms (34). The association between US and mental problems has been well established in Chinese another study (35). Although the association between the US and depressive symptoms has been well established in these studies, however, there is currently not much evidence that uncertainty stress links BCE to depressive symptoms.

The stress sensitization model (36) proposed that stressful life events that occurred in the past year may serve as a trigger in the pathways from childhood experience to adulthood mental disorders (37, 38), and the stress sensitization effect was strong among people with multiple childhood adversities (37). We speculate that BCE could reduce the perception of stress events, and reduce the risk of the US turning into depressive symptoms. Doom et al. also made a point that the stressor will influence the relationship between childhood experiences and current mental health problems (13). To sum up, we hypothesis that BCE can affect depressive symptoms through a mediation pathway of US among undergraduates.

### Potential Role of Family Relationship in Moderating the Association Between BCE and Depressive Symptoms

Family relationship, including child-parent relationship, parent relationship, and family climate, which has been suggested as the main predictors of depressive symptoms among child (39, 40). All of the less warmth, more inter-parental conflict, over-involvement in the family can cause a higher risk of depression (41). Attachment theory proposed that the close relationship between parents and child serves as affective support and a safe base, which could contribute to multiple aspects of psychosocial adaptations (42). This implies that better family relationships could shape a person's beliefs about the acceptability and expression of emotions when they encounter the US, preventing the US switch to depressive symptoms (42). In addition, the change of family and household could arouse the relapse into episodes of mental problems which recovered from stress events before (43). This suggests that the family relationship may have a protective effect prevent the US switch to mental problems. The family relationship can also provide a foundation for creating better BCE (44), which implies that family relationships could strengthen the protective effect for depressive symptoms. Therefore, we assumed that family relationship moderates the linkage between uncertainty stress and depressive symptoms which mediate the association between BCE and depressive symptoms.

**Figure 1:**
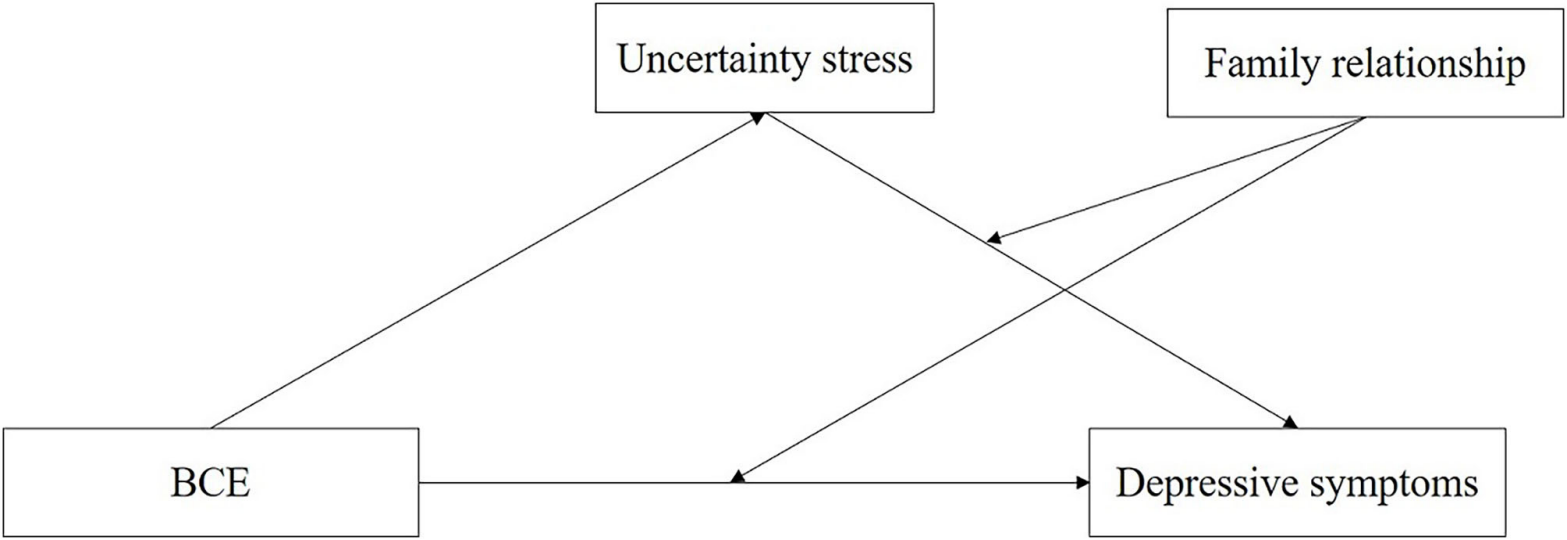
The hypothesis model of the relationships between BCE, uncertainty stress, family relationship, and depressive symptoms.

To date, none of the studies constructed an integrative moderated mediation model to explore the underlying mechanism between BCE, US, and depressive symptoms, as well as the effects of family relationships. To bridge these knowledge gaps, our research investigated BCE, US, and depressive symptoms in a random sample of undergraduates in China, and tested whether: (1) US mediate the link between BCE and depressive symptoms; (2) Family relationship moderates the link between BCE and US, as well as the link between the US and depressive symptoms (See Figure 1). Basic characteristics and variables that may affect the outcome variables were used as control variables.

## Methods

### Study Design and Data Collection

From March 2021 to May 2021, a cross-sectional study was conducted among undergraduate students in three cities (Xuzhou, Nanjing, and Wuhan), China, by using an online survey platform (www.wjx.com). Undergraduate students were randomly selected from a total of 25 universities by using a stratified multistage cluster sampling method. First, a stratified sampling method was used to select schools by taking the school levels as the indicators. A total of 25 universities were selected. In each university, a stratified (according to the majors) random sampling method was used to select the classes, and cluster sampling was then used in each class. A questionnaire guider accepted train before the survey was set in every class. Completion of the questionnaire was voluntary, no incentive was provided, and anonymity was assured. Among 2022 undergraduate students who completed the questionnaire, 201 were excluded because of the not reliable answers or timeout answers (<100 s). Finally, a total of 1,821 participants were included in our study with an effective response rate of 90.06%.

The Ethics Committee of Xuzhou Medical University has reviewed and approved the study protocol.

## Assessment

### Basic Characteristics

Information regarding the participants' gender, age, grade, sibling, ethnicity and residence, parent's marriage and education, living expenses, sexual orientation, and academic performance were asked to understand the characteristics of the participants.

### Benevolent Childhood Experiences

Benevolent childhood experience (BCE) was measured by the BCEs scale (14) which includes 10 items of positive childhood experiences occurring before 18 years old. Items include (1) having at least one safe caregiver, (2) having at least one good friend, (3) having beliefs that gave comfort, (4) enjoying school, (5) having at least one teacher who cared, (6) having good neighbors, (7) having an adult (not a parent/caregiver) who could provide support or advice, (8) having opportunities to have a good time, (9) having a positive self-concept, and (10) having a predictable home routine. Each “Yes” response was scored as a one and a “No” response answer as a 0. A total score of BCEs was summed by 10 items (range = 0–10), and the higher score reflects more positive childhood experiences. The Cronbach's α of the scale was 0.729 in the present study.

### Uncertainty Stress

The US Questionnaire is a measure of uncertainty stress and has shown good reliability and validity (34, 45). The scale consists of four subscales (10 items) including current status uncertainty, social change uncertainty, goal uncertainty, and social value uncertainty. The items were rated on a standard 5-point Linkert rating scale from 0 (no stress) and four (excessive stress). A total stress score was summed by every single item score. A higher score indicates a high level of stress. The Cronbach's α of the scale was 0.951.

### Family Relationship

Family relationship was measured by four questions that individuals self-reported the relationship in family and the family atmosphere. Items include: (1) The relationship with father, (2) The relationship with mother, (3) The relationship between parents, (4) The atmosphere in the family. The scale was rated on a standard 4-point Linkert rating scale from one (very discordant) and four (very harmonious). The score of each four items was summed up to calculate the total score. A higher score indicated a better family relationship. In this current sample, the Cronbach's alpha for the Family relationship was 0.866.

### Depressive Symptoms

Depressive symptoms in the past week were measured by a 10-items questionnaire (Center for Epidemiologic Studies Depression [CESD]-10), which is a short version of the CESD-20. The scale was rated on a Linkert rating scale from 0 (rarely or none of the time, <1 day) and three (all the time, 5 to 7 days). Item five and Item eight are scored inversely. The total score of 10-items is calculated to assess the depressed mood, the higher score represents the higher depressive symptoms (46, 47). The Cronbach's α of the scale was 0.869 in the current study.

### Data Analyses

Descriptive analyses of the participants' demographic characteristics and Spearman's correlation analysis of BCE, US, Family relationship, and depressive symptoms were calculated by using SPSS 25. We used Process version 3.5 (48, 49) based on SPSS 25 (IBM Corporation, Armnok, NY, USA) to test the mediation model, moderation model, and moderated mediation model. We conducted a mediation analysis following Baron and Kenny's approach (50). Bootstrapping method (48) based on 5,000 bootstrap samples was used to estimate the confidence interval (CI) for the indirect effect and assess the significance of estimated indirect effects. A moderation analysis was conducted to test the moderation effect of the family relationships on the link between BCE and depressive symptoms, as well as the link between the US and depressive symptoms. Finally, we performed an integrative moderated mediation analysis to test the role of family relationships in moderating the purposed mediation model. Significant at *P* ≤ 0.05 (two-sided) were included as controls in all statistical analyses. In addition, covariates were controlled in all main analyses, such as gender, age, grade, only-child, nation, residence, parent's marriage, and education, living expenses, sexual orientation, and academic performance.

## Results

### Sociodemographic Characteristics

As is shown in Table 1, a total of 1,821 undergraduates completed the surveys. Of all the participants, most of them are female (69.47%) and ethnic Han (97.09%). The average age was 20.07 and about 82.31% of respondents are in the sophomore year and junior year. About half (50.03%) of participants have a sibling and 56.62% of participants come from urban, while 43.38% come from rural areas. Only 153 (8.4%) participants' parents divorced and a majority of participants' parents have a junior high school or below degree (43.55 and 52.39%, respectively). Most of the participants are heterosexual (89.51%). Regarding academic performance, 82.15% of participants didn't fail the exam within a year while 17.85% of participants failed the exam within a year. Finally, 1,441 (79.13%) respondents spent 1,001–2,000 yuan within a month.

**Table 1:** Sociodemographic characteristics of undergraduate students (*n* = 1,821).

**Characteristics**	**Frequency (Mean)**	**Percentage (SD)**
Age^a^	20.07	1.24
Gender^b^		
Male	556	30.53
Female	1,265	69.47
Grade		
Freshman	279	15.32
Sophomore	831	45.63
Junior	668	36.68
Senior	43	2.36
Only-child		
Yes	910	49.97
No	911	50.03
Ethnicity		
Ethnic Han	1,768	97.09
Ethnic minorities	53	2.91
Residence		
Urban	1,031	56.62
Rural	790	43.38
Parents divorced		
Yes	1,668	91.60
No	153	8.40
Father education		
Junior high school or below	793	43.55
Senior high school	586	32.18
College	421	23.12
Master or doctor	21	1.15
Mother education		
Junior high school or below	954	52.39
Senior high school	532	29.21
College	319	17.52
Master or doctor	16	0.88
Sexual orientation		
Heterosexual	1,630	89.51
Homosexual	40	2.20
Bisexuality	108	5.93
Other	43	2.36
Fail in exam within a year		
Yes	325	17.85
No	1496	82.15
Living expenses (yuan)		
≤ 1000	92	5.05
1001–2000	1441	79.13
2001–3000	227	12.47
≥3000	61	3.35

a *Below data are shown as mean (SD)*.

b *Below data are shown as n (%)*.

### Preliminary Correlation Analyses

Table 2 shows the results of Spearman's correlational analyses, which indicate that BCE was negatively associated with US (***r*** = −0.388, *P* < 0.01) and depressive symptoms (***r*** = −0.461, *P* < 0.01). While the US was positively associated with depressive symptoms (***r*** = 0.621, *P* < 0.01) and negatively associated with family relationships (***r*** = −0.26, *P* < 0.01). Moreover, the family relationships were negatively associated with depressive symptoms (***r*** = −0.316, *P* < 0.01) and positively associated with BCE (***r*** = 0.307, *P* < 0.01). These results support further test of mediation and moderated mediation models while controlling for covariates.

**Table 2:** Correlation between BCE, uncertainty stress, family relationship, and depressive symptoms among adolescents.

**Variables**	**Mean**	**SD**	**1**	**2**	**3**	**4**
1. BCE	18.67	1.78	1.000			
2. Uncertainty stress	23.19	9.03	−0.388^**^	1.000		
3. Family relationship	13.94	2.21	0.307^**^	−0.260^**^	1.000	
4. Depressive symptoms	17.57	5.93	−0.461^**^	0.612^**^	−0.316^**^	1.000

*(1) Date (r) is correlation coefficient:*

** *P < 0.01*.

### Mediation Modeling Analysis

**Figure 2:**
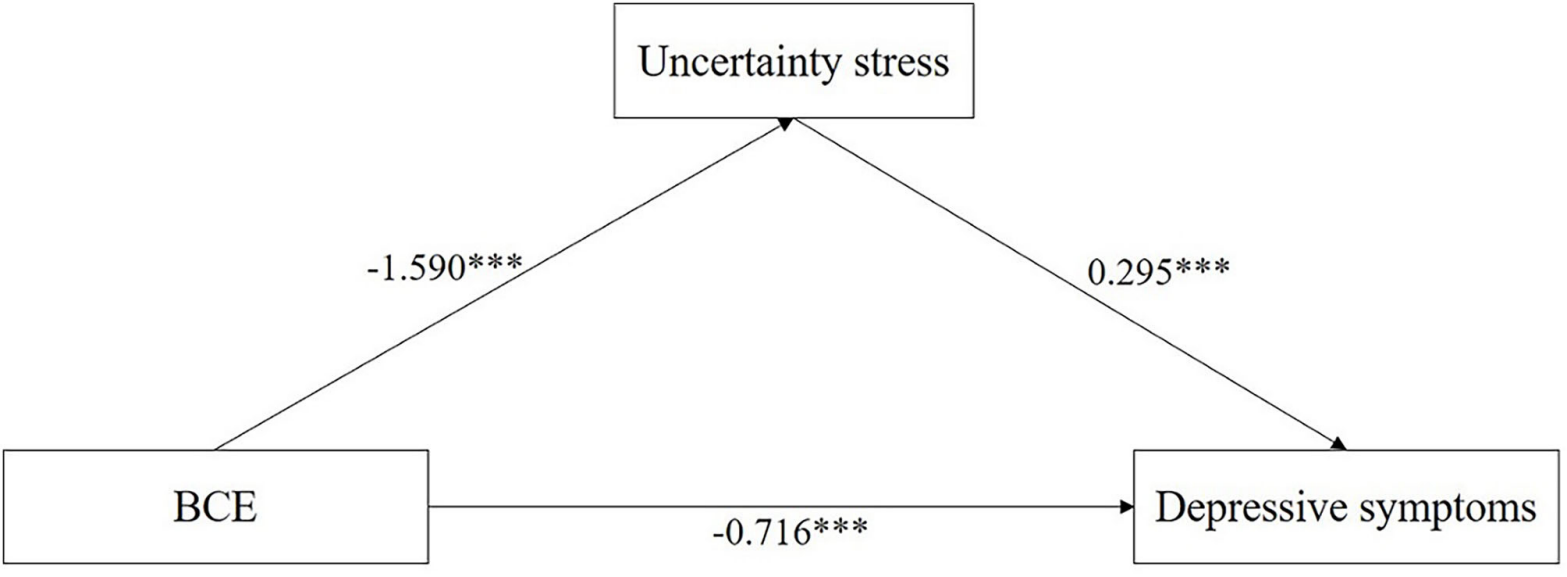
Mediation modeling analysis of the relationship among BCE, uncertainty stress, and depressive symptoms. (1) Covariates controlled in the modeling analysis were Gender, Age, Grade, Only-child, Ethnicity, Residence, Parent's marriage and education, Living expenses, Sexual orientation, Academic performance. (2) *P < 0.05, **P < 0.01, ***P < 0.001.

A mediation model analysis was established to examine the association between BCE, US, and depressive symptoms followed by the results of correlation analyses. Figure 2 illustrated the mediation model, along with standardized path coefficients which indicates that BCE was significantly associated with the US (β = −1.59, *P* < 0.001), and depressive symptoms (β = −0.72, *P* < 0.001) when controlled for covariates. in addition, the results of the non-parametric bootstrapping method suggested that the US has a significant indirect effect in mediating the association between BCE and depressive symptoms (effect = −0.47, 95% bootstrap CI = −0.55, −0.39). The direct effect of BCE on depressive symptoms was also significant (effect = −0.71, 95% bootstrap CI = −0.83, −0.6), indicating a partial mediation of the depressive symptoms when controlled for covariates. The indirect effect of the US accounted for 39.63% of the total variance in depression. These findings are consistent with our hypothesis that the US may play a mediator role in the association between BCE and depressive symptoms.

### Moderation Analysis

**Figure 3:**
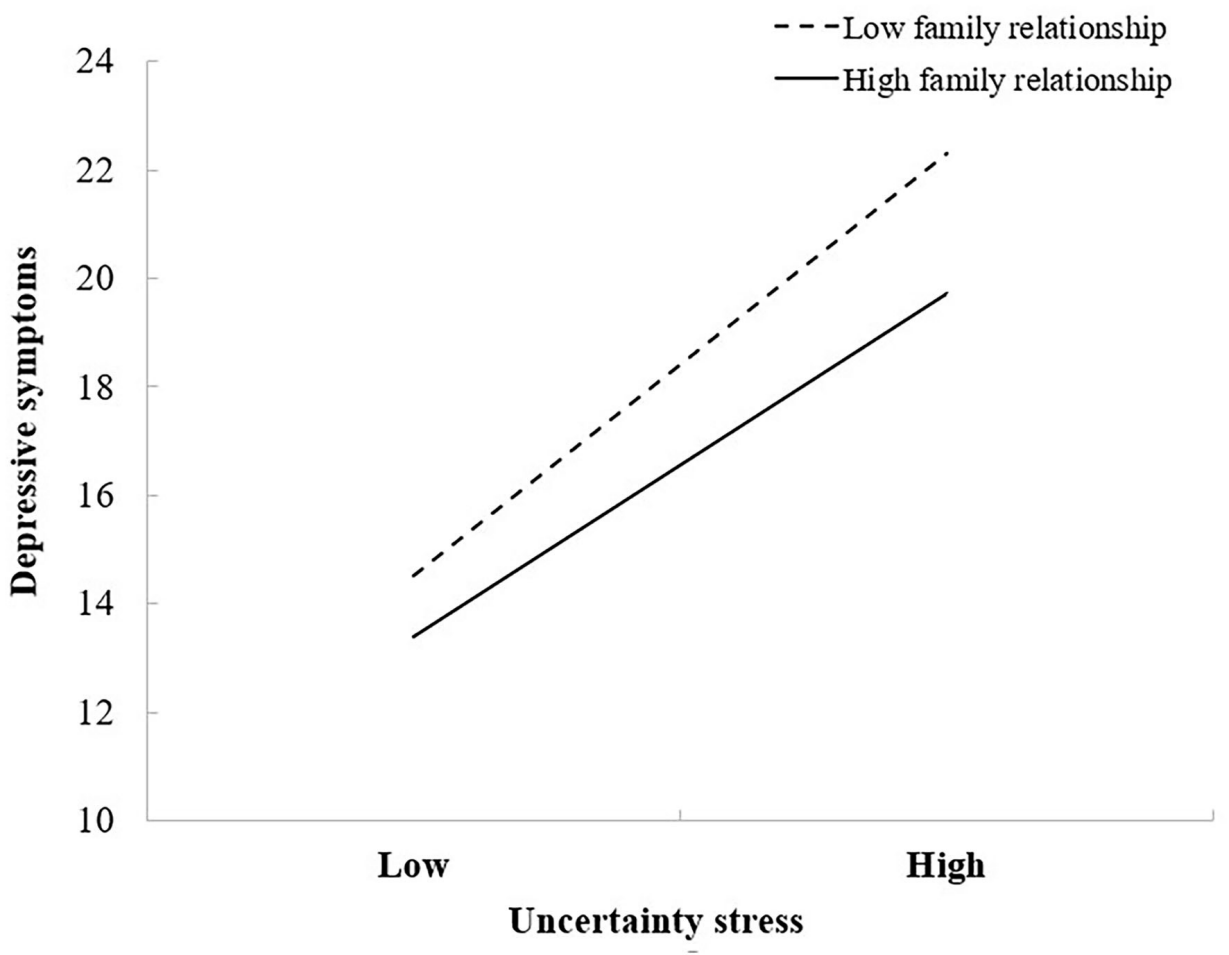
Family relationship moderates the effect of uncertainty stress on depressive symptoms. Covariates controlled in the modeling analysis were the following: Gender, Age, Grade, Only-child, Ethnicity, Residence, Parent's marriage and education, Living expenses, Sexual orientation, and Academic performance.

Only a significant interaction between US and family relationship in predicting depressive symptoms was found in results of moderation analysis (β = −0.019, *P* < 0.001), while the interaction between BCE and family relationship in predicting depressive symptoms was insignificant (β = 0.008, *P* = 0.773). We calculated the simple slopes using the “pick-a-point” approach (51) to examine the changes in the relationship between the US and depressive symptoms with the increase of family relationships. We used the one standard deviation below and above mean to represent the “Low” and “High” levels of family relationships and the US, respectively. Results in Figure 3 illustrates the different slopes associated with different levels of family relationship, as family relationships increased, the effect of US on depressive symptoms decreased: the simple slopes were 0.351 and 0.432 (*P*s < 0.001) at low and high levels of family relationship.

### Moderated Mediation Analysis

**Figure 4:**
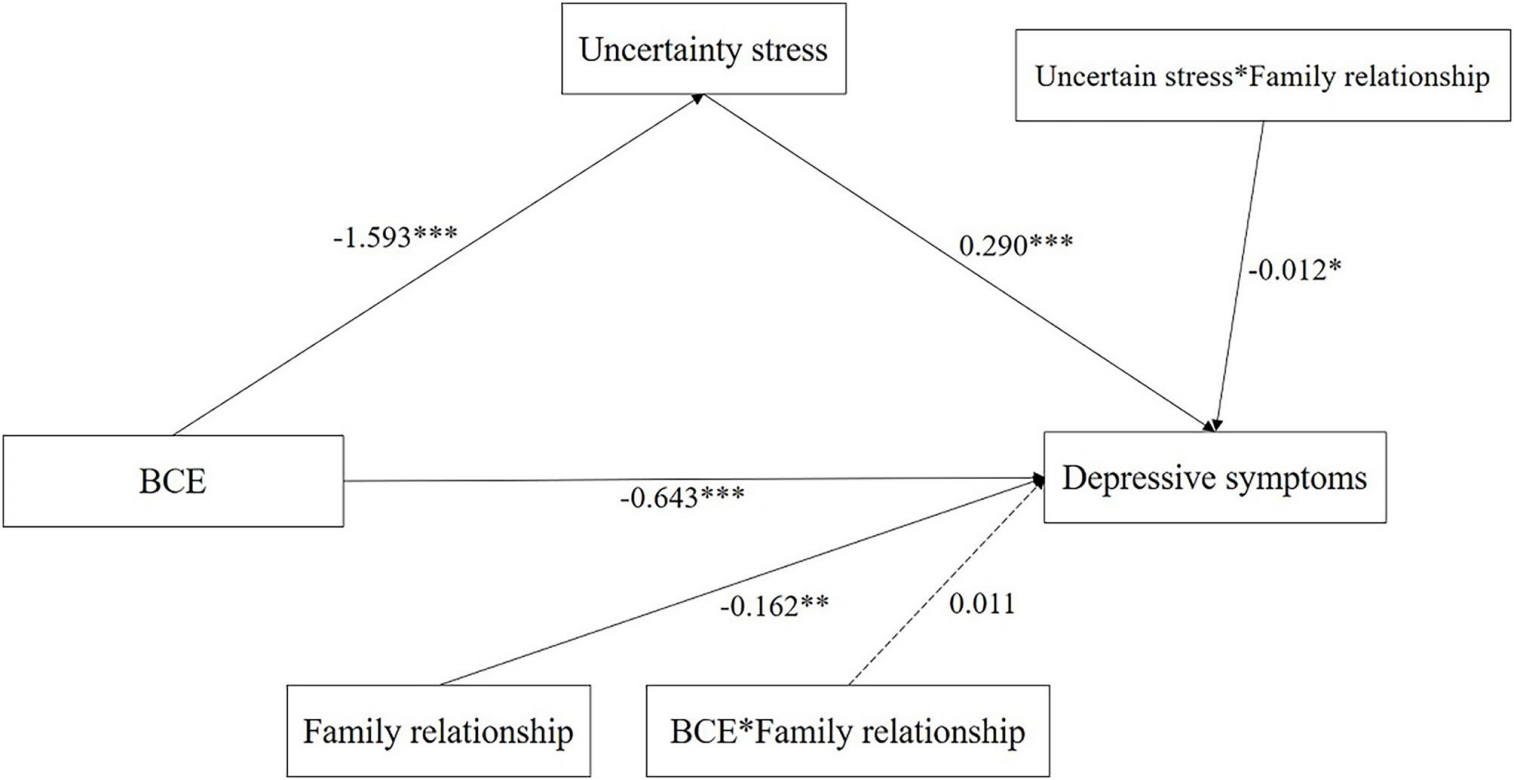
Moderated mediation modeling analysis of the complex relationship among BCE, US, family relationship and depressive symptoms. (1) Covariates controlled in the modeling analysis were the following Gender, Age, Grade, Only-child, Ethnicity, Residence, Parent's marriage and education, Living expenses, Sexual orientation, and Academic performance. (2) *P < 0.05, **P < 0.01, ***P < 0.001. (3) Solid lines represent statistically significant paths. Dotted lines represent non-significant paths.

Table 3 and Figure 4 suggested a significant moderated mediation model: *R*^2^ = 0.577, *F* = 128.927, *P* < 0.001. The results indicate that the interaction term (BCE^*^Family relationship) was not significant (β = 0.011, *P* = 0.659) while the interaction term (US^*^Family relationship) was significant (β = −0.012, *P* = 0.014), which means Family relationship significantly moderated the association between the US and depressive symptoms while the moderation effect of Family relationship on the link between BCE and depressive symptoms was not statistically significant. In addition, the conditional indirect effect of family relationship range low (1 *SD* below the mean) to the high level (1 *SD* above the mean) indicated that the indirect effects of BCE on depressive symptoms through the US were significant across the levels of family relationship. When the family relationship increased from −0.255 (1 *SD* below the mean) to 2.055 (1 *SD* above the mean), the indirect effect of US on depressive symptoms changed from −0.504 to −0.423. Moreover, all of low (β = −0.504, 95% CI −0.603, −0.414), moderate (β = −0.462, 95% CI −0.544, −0.387), and high (β = −0.423, 95% CI −0.513, −0.343) level of family relationship significantly moderated the association between BCE and depressive symptoms.

**Table 3:** Regression coefficients predicting depressive symptoms, family relationship as moderator (moderated mediation model).

**Explanatory variables**	**Uncertainty stress**				**Depressive symptoms**			
	**β**	**SE**	***t***	***P***	**β**	**SE**	***t***	***P***
BCE	−1.593	0.114	−13.939	<0.001	−0.643	0.064	−10.030	<0.001
Family relationship					−0.162	0.048	−3.390	<0.001
Uncertainty stress					0.290	0.012	25.272	<0.001
BCE*Family relationship					0.011	0.025	0.441	0.659
Uncertainty stress *Family relationship					−0.012	0.005	−2.469	0.014
Model summary	*R*^2^ = 0.222, *F* = 34.310, *P* <0.001				*R*^2^ = 0.577, *F* = 128.927, *P* <0.001			
Conditional indirect effect of BCE on depressive symptoms								
**Mediator**	**Family relationship**	**β**	**Boot SE**	**Boot LLCI**	**Boot ULCI**
US	−2.055	−0.504	0.049	−0.603	−0.414
	0.000	−0.462	0.040	−0.544	−0.387
	2.055	−0.423	0.044	−0.513	−0.343

*(1) Covariates controlled in the modeling analysis were the following: Gender, Age, Grade, Only-child, Ethnicity, Residence, Parent's marriage and education, Living expenses, Sexual orientation, and Academic performance. (2) SE, standard error; LLCI, lower levels for confidence interval; ULCI, upper levels for confidence interval*.

## Discussion

To our knowledge, our research is the first study focusing on the underlying mechanisms of BCE, US, and depressive symptoms. Our study finds that BCE has a negative effect on depressive symptoms, which is consistent with the previous study (19, 21, 52). Besides, our research indicates a partial mediation effect of US from BCE to depressive symptoms. Moreover, the family relationship has a significant moderated mediation effect on the indirect relationship from BCE toward depressive symptoms, while the moderated mediation effect of the family relationship on the direct relationship from BCE toward depressive symptoms is insignificant.

### The US as the Underlying Mechanism for the BCE-Depressive Symptom's Link

As hypothesized, we found BCE was negatively associated with the US, which in turn buffer the depressive symptoms. The mediating effect of the US might be attributed to the following reasons. Coping resources theory proposes that coping with stress must be based on good resources, either individual resources (self-esteem, self-efficacy, etc.) or social resources (social support) (30, 53). BCE can increase the acceptance of adult psychosocial resources (54) and social support (13) and then enhance the ability to manage stress, thus reducing the risk of uncertainty stress (55). Secondly, BCE may promote life meaning and strengthen the ability of self-mastery (56), which can help individuals cope with stress and strain (57–59). What's more, our findings indicate that the increase of US can deteriorate the extent of depressive symptoms (60), which is consistent with the previous study (60). In certain situations, the US may become be an obstacle in school study (61), and arouse irrational action in other situations among undergraduates (62). Both situations can contribute to depressive symptoms of undergraduates (63).

Because the future is inherently uncertain, so human beings are always faced with uncertainty. There is no need to treat all uncertainty as a monster. It is severe uncertainty that needs to be prevented and managed. Individuals should develop confidence, give hope, need information, develop skills to cope with uncertainty, etc. (30). Enhance tolerance of uncertainty has been proved effective in the previous study (64, 65). Therefore, Reflective writing (66), Mindfulness-based interventions (67) are needed to raise the level of tolerance of uncertainty.

### Increase Family Relationship as a Potential Intervention Component

One of the most important findings in our study is that family relationships play a moderator in the mediation pathway from BCE to depressive symptoms, which suggests a new way to intervene and reduce the risk of depressive symptoms. Firstly, the better family relationships play as a foundation of BCE (44), which can promote the development of family-related BCE, increasing the protective effect of BCE on the US (55). Secondly, family is one of the most important resources of social support (68) and family resilience (60), the better relationship in the family often represents the higher family social support and family resilience, which can both enhance their BCE and enable them to flourish with warmth, support, and cohesion by successfully coping with the US (60). The attachment theory indicated that one's emotional needs can be satisfied by warm and supportive parenting and a positive family atmosphere, leading to less preoccupation with one's negative mild mental problems (69). Less preoccupation with the US can avoid the US switching to more serious psychological problems (depression, suicidal ideation) effectively (69).

In addition, a better family context is helpful to improve the adjustment of the individual (internalizing, externalizing, social competence) and develop emotion regulation (70), which can enable students to make friends, develop good community and school relations, thus developing good BCE. The ability emotion regulation developed by a good family relationship can help students respond to the US and other emotional experiences in a socially appropriate, adaptive, and flexible manner (71–73). Moreover, adolescents are tending to study the emotion regulation from parents' emotional displays and interactions. The parents' emotional profiles implicitly teach children which emotions are acceptable (74). For example, if parents often display negative emotions in the family, children may perform inappropriate emotional responses when confront with stressful events, which may contribute to depressive symptoms (75). This also highlights the good family relationship can help to ease depressive symptoms for college students.

Although undergraduates mostly leave their families and live with their classmates, however, attachment theory also pointed out that the security, adaptations, and maturity fostered by their parents are more dependent on the role of their parents “competent allies” role and less on their actual presence (76, 77). In other words, the ability of environment adaption fostered by positive family relationships continues to exist when they leave their parents.

With the family structures becoming increasingly complex, at present, less than half of children lived in the family units which used to be the norm in the past, such as two-parent, married, biological parents (78, 79). This suggests that parents should provide a complete family to children. Moreover, positive engagement in family interactions (80) and improving family communication (81) are also helpful to create a positive family relationship.

## Limitations

Some limitations should be noted in our study. Firstly, we can't judge the causality between variables because of the cross-section study. Secondly, the samples were collected from three provinces, China, thus the representativeness of samples is questionable. Also, the self-report questionnaire could exist the bias in this study. Finally, our study only explored the effect of family factors and personal level factors toward depressive symptoms whereas the sociocultural factors may also influence the depressive symptoms of undergraduates. In the future study, we would consider sociocultural factors such as school-related factors and social support in predicting depressive symptoms.

## Conclusions

In summary, the findings of our study provide information not only on the mediate mechanism of how BCE eases depressive symptoms through the US but also the protective mechanism of how the family relationship can buffer the impact of the US on depressive symptoms among undergraduates. Our findings suggest that enhance tolerance of uncertainty and improving family relationships to protect undergraduates from depressive symptoms.

## Data Availability Statement

The raw data supporting the conclusions of this article will be made available by the authors, without undue reservation.

## Ethics Statement

The studies involving human participants were reviewed and approved by Ethics Committee of Xuzhou medical University. Written informed consent to participate in this study was provided by the participants' legal guardian/next of kin.

## Author Contributions

WW and HH: conceptualization, methodology. CZ, HH, and YZ: data curation, writing-original draft preparation. HH, CH, YP, and JW: supervision, validation. WW and XG: writing-reviewing and editing. WW: revising. All authors contributed to the article and approved the submitted version.

## Funding

This work was supported by the National Natural Science Foundation of China [Grant Number: 82003484] and Universities' philosophy and social science researches in Jiangsu Province [2020SJA1053], Natural Science Foundation for Colleges Universities in Jiangsu Province [20KJB330005].

## Conflict of Interest

The authors declare that the research was conducted in the absence of any commercial or financial relationships that could be construed as a potential conflict of interest.

## Publisher's Note

All claims expressed in this article are solely those of the authors and do not necessarily represent those of their affiliated organizations, or those of the publisher, the editors and the reviewers. Any product that may be evaluated in this article, or claim that may be made by its manufacturer, is not guaranteed or endorsed by the publisher.

## References

[1] Kessler RC Berglund P Demler O Jin R Koretz D Merikangas KR. The epidemiology of major depressive disorder: results from the national comorbidity survey replication (NCS-R). JAMA. (2003) 289:3095–105. 10.1001/jama.289.23.3095 12813115

[2] Ibrahim AK Kelly SJ Adams CE Glazebrook C. A systematic review of studies of depression prevalence in university students. J Psychiatr Res. (2013) 47:391–400. 10.1016/j.jpsychires.2012.11.015 23260171

[3] Gao L Xie Y Jia C Wang W. Prevalence of depression among Chinese university students: a systematic review and meta-analysis. Sci Rep. (2020) 10:15897. 10.1038/s41598-020-72998-1 32985593 PMC7522998

[4] Lei XY Xiao LM Liu YN Li YM. Prevalence of depression among Chinese University students: a meta-analysis. PLoS ONE. (2016) 11:e0153454. 10.1371/journal.pone.0153454 27070790 PMC4829172

[5] Jiang CX Li ZZ Chen P Chen LZ. Prevalence of depression among college-goers in mainland China: a methodical evaluation and meta-analysis. Medicine. (2015) 94:e2071. 10.1097/MD.0000000000002071 26683916 PMC5058888

[6] Pillay N Ramlall S Burns JK. Spirituality, depression and quality of life in medical students in KwaZulu-Natal. SAJP. (2016) 22:731. 10.4102/sajpsychiatry.v22i1.731 30263152 PMC6138166

[7] Wang YH Shi ZT Luo QY. Association of depressive symptoms and suicidal ideation among university students in China: a systematic review and meta-analysis. Medicine. (2017) 96:e6476. 10.1097/MD.0000000000006476 28353586 PMC5380270

[8] Felitti VJ Anda RF Nordenberg D Williamson DF Spitz AM Edwards V. Relationship of childhood abuse and household dysfunction to many of the leading causes of death in adults. ACE. (1998) 14:245–58. 10.1016/S0749-3797(98)00017-8 9635069

[9] Tracy M Salo M Slopen N Udo T Appleton AA. Trajectories of childhood adversity and the risk of depression in young adulthood: results from the Avon longitudinal study of parents and children. Depress Anxiety. (2019) 36:596–606. 10.1002/da.22887 30884010 PMC6602824

[10] Tsehay M Necho M Mekonnen W. The role of adverse childhood experience on depression symptom, prevalence, and severity among school going adolescents. Depress Res Treat. (2020) 2020:5951792. 10.1155/2020/5951792 32257437 PMC7104267

[11] Bernet CZ Stein MB. Relationship of childhood maltreatment to the onset and course of major depression in adulthood. Depress Anxiety. (1999) 9:169–74. 10431682

[12] Narayan AJ Lieberman AF Masten AS. Intergenerational transmission and prevention of adverse childhood experiences (ACEs). Clin Psychol Rev. (2021) 85:101997. 10.1016/j.cpr.2021.101997 33689982

[13] Doom JR Seok D Narayan AJ Fox KR. Adverse and benevolent childhood experiences predict mental health during the COVID-19 pandemic. Adversity and resilience science. (2021) 23:1–12. 10.31234/osf.io/vr5jd 33907733 PMC8062213

[14] Narayan AJ Rivera LM Bernstein RE Harris WW Lieberman AF. Positive childhood experiences predict less psychopathology and stress in pregnant women with childhood adversity: a pilot study of the benevolent childhood experiences (BCEs) scale. Child Abuse Negl. (2018) 78:19–30. 10.1016/j.chiabu.2017.09.022 28992958

[15] Slopen N Chen Y Guida JL Albert MA Williams DR. Positive childhood experiences and ideal cardiovascular health in midlife: associations and mediators. Preventive medicine. (2017) 97:72–9. 10.1016/j.ypmed.2017.01.002 28087467 PMC5430499

[16] Lee H. Schafer M. Are positive childhood experiences linked to better cognitive functioning in later life?: examining the role of life course pathways. J Aging Health. (2021) 33:217–26. 10.1177/0898264320972547 33228449 PMC7906946

[17] Crouch E Radcliff E Merrell MA Bennett KJ. Rural-urban differences in positive childhood experiences across a national sample. J rural health. (2021) 37:495–503. 10.1111/jrh.12493 32639648

[18] Wang YP. Intergenerational rearing in the vision of brain science and its enlightenment to education. J Chinese Educ. (2014) 44–7. [In Chinese].

[19] Bethell C Jones J Gombojav N Linkenbach J Sege R. Positive childhood experiences and adult mental and relational health in a statewide sample: associations across adverse childhood experiences levels. JAMA Pediatr. (2019) 173:e193007. 10.1001/jamapediatrics.2019.3007 31498386 PMC6735495

[20] Andersson M. AJJHSB. Chronic disease at midlife: do parent-child bonds modify the effect of childhood SES? J Health Soc Behav. (2016) 57:373–89. 10.1177/0022146516661596 27601411

[21] Crandall A Miller JR Cheung A Novilla LK Glade R Novilla MLB. ACEs and counter-ACEs: how positive and negative childhood experiences influence adult health. Child Abuse Negl. (2019) 96:104089. 10.1016/j.chiabu.2019.104089 31362100

[22] Yang T Yang XY Yu L Cottrell RR Jiang S. Individual and regional association between socioeconomic status and uncertainty stress, and life stress: a representative nationwide study of China. Int J Equity Health. (2017) 16:118. 10.1186/s12939-017-0618-7 28679409 PMC5498910

[23] Holland D Wheeler HJMSR. College Student Stress and Mental Health: Examination of Stigmatic Views on Mental Health Counseling. (2016). p. 30.

[24] Peng S Yang T Rockett IRH. Life stress and uncertainty stress: which is more associated with unintentional injury? Psychol Health Med. (2020) 25:774–80. 10.1080/13548506.2019.1687913 31684773

[25] Yang T Barnett R Fan Y Li L. The effect of urban green space on uncertainty stress and life stress: a nationwide study of university students in China. Health Place. (2019) 59:102199. 10.1016/j.healthplace.2019.102199 31514059

[26] Ye L Zhang X Geng J. Demographic transition and economic growth: evidence from China and United States. Int J Health Plann Manage. (2020) 35:e1–e11. 10.1002/hpm.2911 31694067

[27] Scharping TJJoCC. Abolishing the one-child policy: stages, issues and the political process. J Contemp China. (2018). 28:1–21. 10.1080/10670564.2018.1542217

[28] Ye L Wu AM Yang XJSSEP. University Enrolment Expansion and Returns to Higher Education: Evidence from China (2018).

[29] Mirowsky J. Ross CEJJoH, Behavior S. Control or defense? depression and the sense of control over good and bad outcomes. J Health Soc Behav. (1990) 31:71–86. 10.2307/2137046 2313078

[30] Yang TZ. Health Behavior Theory and Research, Beijing: People's medical publishing house (2007).

[31] Ma Q Qiu W Fu H Sun X. Uncertain is worse: modulation of anxiety on pain anticipation by intensity uncertainty: evidence from the ERP study. Neuroreport. (2018) 29:1023–9. 10.1097/WNR.0000000000001061 29846299

[32] McEvoy PM Mahoney AE. Achieving certainty about the structure of intolerance of uncertainty in a treatment-seeking sample with anxiety and depression. J Anxiety Disord. (2011) 25:112–22. 10.1016/j.janxdis.2010.08.010 20828984

[33] Wu D Rockett IR Yang T Feng X Jiang S Yu L. Deliberate self-harm among Chinese medical students: A population-based study. J Affect Disord. (2016) 202:137–44. 10.1016/j.jad.2016.05.030 27262635

[34] Wu D Yu L Yang T Cottrell R Peng S Guo W. The impacts of uncertainty stress on mental disorders of chinese college students: evidence from a nationwide study. Front Psychol. (2020) 11:243. 10.3389/fpsyg.2020.00243 32210868 PMC7075936

[35] Chen TY Kao CW Cheng SM Chang YC. Uncertainty and depressive symptoms as mediators of quality of life in patients with heart failure. PLoS ONE. (2018) 13:e0205953. 10.1371/journal.pone.0205953 30427855 PMC6235604

[36] Hammen Constance Henry Consulting RJJo Psychology C. Depression and sensitization to stressors among young Women as a function of childhood adversity. J Consult Clin Psychol. (2000) 68:782–7. 11068964

[37] Mclaughlin KA Conron KJ Koenen KC. Gilman SEJCP. Childhood adversity, adult stressful life events, and risk of past-year psychiatric disorder: a test of the stress sensitization hypothesis in a population-based sample of adults. Psychol Med. (2010) 40:1647–58. 10.1017/S0033291709992121 20018126 PMC2891275

[38] Zhou Q Yin Z Wu W Li NJIH. Childhood familial environment and adulthood depression: evidence from a Chinese population-based study. International health. (2019) 12:299–316. 10.1093/inthealth/ihz084 31642909 PMC7322201

[39] Lin JD Tung HJ Hsieh YH Lin FG. Interactive effects of delayed bedtime and family-associated factors on depression in elementary school children. Research in developmental disabilities. (2011) 32:2036–44. 10.1016/j.ridd.2011.08.011 21985986

[40] Kim K Birditt KS Zarit SH Fingerman KL. Typology of parent-child ties within families: Associations with psychological well-being. JFP. (2020) 34:448–58. 10.1037/fam0000595 31599601 PMC7145731

[41] Yap MB Pilkington PD Ryan SM Jorm AF. Parental factors associated with depression and anxiety in young people: a systematic review and meta-analysis. J Affect Disord. (2014) 156:8–23. 10.1016/j.jad.2013.11.007 24308895

[42] Freed RD Rubenstein LM Daryanani I Olino TM Alloy LB. The relationship between family functioning and adolescent depressive symptoms: the role of emotional clarity. J Youth Adolesc. (2016) 45:505–19. 10.1007/s10964-016-0429-y 26832726 PMC4769177

[43] Francis JL Moitra E Dyck I Keller MB. The impact of stressful life events on relapse of generalized anxiety disorder. Depress Anxiety. (2012) 29:386–91. 10.1002/da.20919 22431499 PMC3667630

[44] Daines CL Hansen D Novilla MLB Crandall A. Effects of positive and negative childhood experiences on adult family health. BMC Public Health. (2021) 21:651. 10.1186/s12889-021-10732-w 33820532 PMC8022401

[45] Yang TZ. Huang H-T. An epidemiological study on stress among urban residents in social transition period. Zhonghua Liu Xing Bing Xue Za Zhi. (2003) 24:760. [In Chinese]. 14521764

[46] Radloff LSJAPM. The CES-D Scale. (2016) 1:385–401. 10.1177/014662167700100306

[47] Salazar-Pousada D Arroyo D Hidalgo L Pérez-López FR Chedraui P. Depressive symptoms and resilience among pregnant adolescents: a case-control study. Obstet Gynecol Int. (2010) 2010:952493. 10.1155/2010/952493 21461335 PMC3065659

[48] Preacher KJ. Hayes AFJBRM. Asymptotic and resampling strategies for assessing and comparing indirect effects in multiple mediator models. (2008) 40:879–91. 10.3758/BRM.40.3.879 18697684

[49] Preacher KJ Rucker DD Hayes AF. Addressing Moderated Mediation Hypotheses: Theory, Methods, and Prescriptions. Multivariate Behav Res. (2007) 42:185–227. 10.1080/00273170701341316 26821081

[50] Baron RM Kenny DAJC. Hall. The moderator-mediator variable distinction in social psychological research: conceptual, strategic, and statistical considerations. (1986) 51:1173–82. 10.1037/0022-3514.51.6.1173 3806354

[51] Preacher KJ Curran PJ. Bauer DJJJoE, Statistics B. Computational tools for probing interactions in multiple linear regression, multilevel modeling, and latent curve analysis. (2006) 31:437–48. 10.3102/10769986031004437

[52] Zhang L Fang J Zhang D Wan Y Gong C Su P. Poly-victimization and psychopathological symptoms in adolescence: examining the potential buffering effect of positive childhood experiences. J Affect Disord. (2021) 282:1308–14. 10.1016/j.jad.2021.01.011 33601709

[53] Taylor SE Stanton AL. Coping resources, coping processes, and mental health. Annu Rev Clin Psychol. (2007) 3:377–401. 10.1146/annurev.clinpsy.3.022806.091520 17716061

[54] Ferraro KF Schafer MH. Visions of the life course: risks, resources, and vulnerability. Res Hum Dev. (2017) 14:88–93. 10.1080/15427609.2016.1268895 28993720 PMC5630178

[55] Braun-Lewensohn O Mayer CH. Salutogenesis and coping: ways to overcome stress and conflict. International Journal of Environmental Research and Public Health. (2020) 17. 10.3390/ijerph17186667 32933161 PMC7557564

[56] Biglan A Flay BR Embry DD Sandler IN. The critical role of nurturing environments for promoting human well-being. The American Psychologist. (2012). 67:257–71 10.1037/a0026796 22583340 PMC3621015

[57] Miller L Lachman ME. Cognitive Performance and the Role of Control Beliefs in Midlife. Aging, Neuropsychology, and Cognition. (2000) 7:69–85. 10.1076/1382-5585(200006)7:2;1-U;FT069

[58] Wang D Jiang Q Yang Z Choi JK. The longitudinal influences of adverse childhood experiences and positive childhood experiences at family, school, and neighborhood on adolescent depression and anxiety. J Affect Disord. (2021) 292:542–51. 10.1016/j.jad.2021.05.108 34147966

[59] Crandall A Broadbent E Stanfill M Magnusson BM Novilla MLB Hanson CL. The influence of adverse and advantageous childhood experiences during adolescence on young adult health. Child Abuse Negl. (2020) 108:104644. 10.1016/j.chiabu.2020.104644 32795716

[60] Simpkin AL Khan A West DC Garcia BM Sectish TC Spector ND. stress from uncertainty and resilience among depressed and burned out residents: a cross-sectional study. Acad Pediatr. (2018) 18:698–704. 10.1016/j.acap.2018.03.002 29524616

[61] Scott A Sudlow M Shaw E Fisher J. Medical education, simulation and uncertainty. Clin Teach. (2020) 17:497–502. 10.1111/tct.13119 31903672

[62] Lin D Friedman DB Qiao S Tam CC Li X Li X. Information uncertainty: a correlate for acute stress disorder during the COVID-19 outbreak in China. BMC Public Health. (2020) 20:1867. 10.1186/s12889-020-09952-3 33287780 PMC7719728

[63] Teshome Hambisa M Derese A Abdeta T. Depressive symptoms among haramaya university students in ethiopia: a cross-sectional study. Depress Res Treat. (2020) 2020:5027918. 10.1155/2020/5027918 32099677 PMC7013291

[64] Palitz SA Rifkin LS Norris LA Knepley M Fleischer NJ Steinberg L. Kendall PC. But what will the results be?: Learning to tolerate uncertainty is associated with treatment-produced gains. Journal of anxiety disorders. (2019) 68:102146. 10.1016/j.janxdis.2019.102146 31669785

[65] Talkovsky AM Norton PJ. Intolerance of uncertainty and transdiagnostic group cognitive behavioral therapy for anxiety. J Anxiety Disord. (2016) 41:108–14. 10.1016/j.janxdis.2016.05.002 27212226

[66] Nevalainen MK Mantyranta T Pitkala KH. Facing uncertainty as a medical student–a qualitative study of their reflective learning diaries and writings on specific themes during the first clinical year. Patient Educ Couns. (2010) 78:218–23. 10.1016/j.pec.2009.07.011 19767167

[67] Papenfuss I Lommen MJJ Grillon C Balderston NL Ostafin BD. Responding to uncertain threat: A potential mediator for the effect of mindfulness on anxiety. J Anxiety Disord. (2021) 77:102332. 10.1016/j.janxdis.2020.102332 33160276

[68] Lei X Kantor J. Social support and family functioning in Chinese families of children with autism spectrum disorder. International journal of environmental research and public health. (2021). 18. 10.3390/ijerph18073504 33800586 PMC8037478

[69] Review by: Anthony Sociology GJBJo. Attachment and Loss, Volume I: Attachment by John Bowlby (1970).

[70] Morris AS Silk JS Steinberg L Myers SS Robinson LR. The role of the family context in the development of emotion regulation. Social development. (2007) 16:361–88. 10.1111/j.1467-9507.2007.00389.x 19756175 PMC2743505

[71] Cole PM Michel MK Teti LO. The development of emotion regulation and dysregulation: a clinical perspective. Monogr Soc Res Child Dev. (1994) 59:73–100. 10.2307/1166139 7984169

[72] Eisenberg N Morris AS. Children's emotion-related regulation. Adv Child Dev Behav. (2002) 30:189–229. 10.1016/S0065-2407(02)80042-8 12402675

[73] Lee H Ahn JK Kwon JH. Effects of self-image on anxiety, judgement bias and emotion regulation in social anxiety disorder. Behav Cogn Psychother. (2019) 47:81–94. 10.1017/S135246581800022X 29692272

[74] Parke RDJM-PQ. Progress paradigms, and unresolved problems: a commentary on recent advances in our understanding of children's emotions. APA PsycNet. (1994) 40:157–69.

[75] Denham SA Mitchell-Copeland J Strandberg K Auerbach S Blair KJM. Emotion parental contributions to preschoolers' emotional competence: direct and indirect effects. (1997) 21:65–86. 10.1023/A:1024426431247

[76] Weiss RS. Attachment in Adult Life (1982).

[77] Armsden GC. Greenberg MT. The inventory of parent and peer attachment: individual differences and their relationship to psychological well-being in adolescence. J Youth Adolesc. (1987) 16:427–54. 10.1007/BF02202939 24277469

[78] Andersson GJMWP. Children's experience of family disruption and family formation: evidence from 16 FFS countries. MPIDR Working Papers WP-2001-028, Max Planck Institute for Demographic Research, Rostock, Germany. (2001).

[79] Cherlin AJ. Seltzer JAJTAotAAoP, Science S. Family Complexity, the Family Safety Net, and Public Policy. Ann Am Acad Pol Soc Sci. (2014) 654:231–9. 10.1177/0002716214530854 26478579 PMC4605537

[80] Ackerman RA Kashy DA Donnellan MB Conger RD. Positive-engagement behaviors in observed family interactions: a social relations perspective. JFP. (2011) 25:719–30. 10.1037/a0025288 21875194 PMC3292257

[81] Riesch SK Anderson LS Krueger HA. Parent-child communication processes: preventing children's health-risk behavior. JSPN. (2006) 11:41–56. 10.1111/j.1744-6155.2006.00042.x 16409505

